# Dietary silver nanoparticles can disturb the gut microbiota in mice

**DOI:** 10.1186/s12989-016-0149-1

**Published:** 2016-07-08

**Authors:** Sybille van den Brule, Jérôme Ambroise, Hélène Lecloux, Clément Levard, Romain Soulas, Pieter-Jan De Temmerman, Mihaly Palmai-Pallag, Etienne Marbaix, Dominique Lison

**Affiliations:** 1 Louvain centre for Toxicology and Applied Pharmacology, Institut de Recherche Expérimentale et Clinique, Université catholique de Louvain, Avenue E. Mounier 52 - bte B1.52.12, 1200 Brussels, Belgium; 2 Centre de Technologies Moléculaires Appliquées, Institut de Recherche Expérimentale et Clinique, Université catholique de Louvain, Clos Chapelle-aux-champs 30 bte B1.30.24, 1200 Brussels, Belgium; 3 CEREGE, Aix Marseille Université, CNRS, IRD, UM34, UMR 7330, Europole de l’arbois - BP 80, 13545 Aix en Provence, France; 4 CEA LITEN Grenoble, 17 Rue des Martyrs, 38054 GRENOBLE - CEDEX 9, France; 5 Electron Microscopy Unit, Veterinary and Agrochemical Research Centre (CODA-CERVA), Groeselenberg 99, 1180 Brussels, Belgium; 6 De Duve Institute, Université catholique de Louvain, Avenue Hippocrate 75 - bte B1.75.02, 1200 Brussels, Belgium

**Keywords:** Nanomaterials, Toxicity, Bacteria, Dysbiosis, Sulfidation, Food, NGS

## Abstract

**Background:**

Humans are increasingly exposed via the diet to Ag nanoparticles (NP) used in the food industry. Because of their anti-bacterial activity, ingested Ag NP might disturb the gut microbiota that is essential for local and systemic homeostasis. We explored here the possible impact of dietary Ag NP on the gut microbiota in mice at doses relevant for currently estimated human intake.

**Methods:**

Mice were orally exposed to food (pellets) supplemented with increasing doses of Ag NP (0, 46, 460 or 4600 ppb) during 28 d. Body weight, systemic inflammation and gut integrity were investigated to determine overall toxicity, and feces DNA collected from the gut were analyzed by Next Generation Sequencing (NGS) to assess the effect of Ag NP on the bacterial population. Ag NP were characterized alone and in the supplemented pellets by scanning transmission electron microscopy (STEM) and energy dispersive X-ray analysis (EDX).

**Results:**

No overall toxicity was recorded in mice exposed to Ag NP. Ag NP disturbed bacterial evenness (α-diversity) and populations (β-diversity) in a dose-dependent manner. Ag NP increased the ratio between Firmicutes (F) and Bacteroidetes (B) phyla. At the family level, Lachnospiraceae and the S24-7 family mainly accounted for the increase in Firmicutes and decrease in Bacteroidetes, respectively. Similar effects were not observed in mice identically exposed to the same batch of Ag NP-supplemented pellets aged during 4 or 8 months and the F/B ratio was less or not modified. Analysis of Ag NP-supplemented pellets showed that freshly prepared pellets released Ag ions faster than aged pellets. STEM-EDX analysis also showed that Ag sulfidation occurred in aged Ag NP-supplemented pellets.

**Conclusions:**

Our data indicate that oral exposure to human relevant doses of Ag NP can induce microbial alterations in the gut. The bacterial disturbances recorded after Ag NP are similar to those reported in metabolic and inflammatory diseases, such as obesity. It also highlights that Ag NP aging in food, and more specifically sulfidation, can reduce the effects of Ag NP on the microbiota by limiting the release of toxic Ag ions.

**Electronic supplementary material:**

The online version of this article (doi:10.1186/s12989-016-0149-1) contains supplementary material, which is available to authorized users.

## Background

Nanomaterials (NM) are widely used in the food industry to take advantage of their very attractive physicochemical properties for improving food taste and texture, increasing nutrient bioavailability, encapsulating components or controlling the release of flavors. Another interesting and growing application of NM is for covering food processing surfaces and packaging, mostly to improve their mechanical and antimicrobial properties [1–3].

Silver nanoparticles (Ag NP) are specifically used for their antimicrobial properties. More than 400 consumer products containing Ag NP are currently inventoried by the project on Emerging Nanotechnologies [4]. Humans and the environment are, therefore, widely and increasingly exposed to Ag NP. Oral exposure is one of the main routes of human exposure to Ag NP via their incorporation in food products or in coating components from food processing machine, as well as in plastic food packaging to prevent bacterial proliferation. In 2009, the human dietary intake of silver was estimated at 70–90 μg/day but this assessment probably underestimates current exposure levels because of the increasing uses of Ag NP by the food industry in recent years [5].

Ag NP show limited toxicity on eukaryotic cells, primarily resulting from increased intracellular levels of reactive oxygen species and oxidative stress [6]. Animal studies showed that Ag NP were detected in most organs after oral exposures [7–9]. The in vivo impact of oral exposure to Ag NP has been investigated in weaned pigs, rats and mice but the results are not consistent. After 14 days of exposure to Ag NP in diet, no effects were detected in the ileal mucosa of pigs but a significant increase of body weight (b.w.) was observed [10]. In contrast, studies in mice and rats noted that Ag NP reduced animal growth rate and affected intestinal microvilli and liver [9, 11, 12], while others did not find any influence on the b.w. [13–15]. Park detected toxicity and inflammation in liver and kidney but no difference in b.w. [16]. The no observed adverse effect level (NOAEL) for systemic effects of Ag NP administered orally is 30 mg/kg [11]. On the other hand, Ag NP demonstrate strong antibacterial functions mainly mediated by the release of silver ions [17]. Nano-silver is an effective bactericidal agent against Gram-negative and Gram-positive strains, its activity depending on the thickness of the peptidoglycan layer of the bacterial wall. Silver ions can bind to negatively charged bacterial surface and disturb its integrity. Ag NP and ions can enter bacterial cells and interfere with the respiratory chain and phosphate uptake. Impaired DNA replication or protein modifications as well as strong interactions with cytoplasmic and membrane thiol-containing proteins are also mentioned as critical effects of Ag NP in bacteria.

Because of their anti-bacterial activity, ingested Ag NP could disturb the gut microbiota that is now considered as an entire metabolic organ with numerous physio(patho)logical functions [18]. This symbiotic ecosystem composed of more than 100 trillion bacteria is essential for intestine maturation, local angiogenesis, regulation of enterocyte gene expression, vitamin synthesis and homeostasis of innate and adaptive immunity [19, 20]. It is vital that a crosstalk between the host and the microbiota is set up just after birth to allow mutual assistance relationships and to help the host developing and maintaining a healthy status [21]. The gut microbiota composition shows intra- and inter- species variations. It continuously evolves from birth to adulthood and an alteration in the bacterial balance can occur following changes in the host environment, diet or circadian rhythm [22–24]. A number of pathological conditions, including asthma, diabetes, obesity and inflammatory bowel disease are associated with alterations of the gut microbiota composition and functions, also called dysbiosis [25–29].

Because of the increased potential for consumer exposure to Ag NP, it appeared urgent to assess the possible impact on the gut microbiota and on human health. Few studies have investigated this issue and none are conclusive [10, 30–34]. We used Next Generation Sequencing (NGS) to investigate the bacterial diversity of the gut microbiota of mice exposed during 28 d to dietary doses of Ag NP relevant for currently estimated daily human intakes.

## Results and discussion

### Characterization of silver NP

**Fig. 1:**
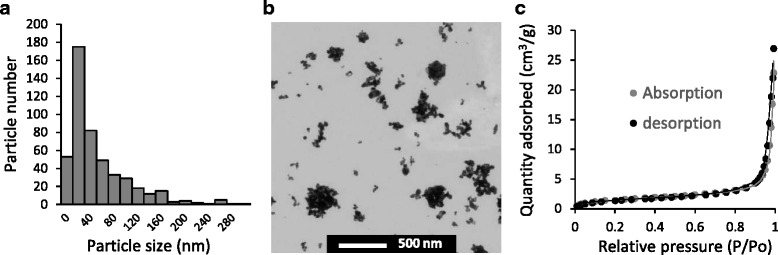
Characteristics of Ag NP. **a** size (diameter) distribution, **b** representative micrograph (TEM analysis), **c** adsorption isotherm (N_2_ physisorption)

Size, surface area and porosity of Ag NP were determined by transmission electron microscopy (TEM) and N_2_ physisorption. TEM analysis revealed a mean area equivalent circular diameter of 55.17 ± 2.67 nm and a right-skewed size distribution shown in Fig. 1a. Individual and agglomerated Ag NP are shown in Fig. 1b. N_2_ physisorption revealed that Ag NP were non porous (porosity 0.027 cm^3^/g) as shown by a typical type-II shape adsorption isotherm (Fig. 1c, see Additional file 1 for further explanation) and had a low specific surface area (5.57 ± 0.08 m^2^/g).

### No overt toxicity in mice orally exposed to Ag NP

Mice were orally exposed to food pellets supplemented with 0, 46, 460 or 4600 ppb Ag NP (μg Ag NP/kg pellet) during 28 d. Considering a 20 g mouse consuming an average of 5 g pellets per day, we aimed to achieve an intake of 0, 11.4, 114 and 1140 μg Ag NP/kg b.w./d (target intake, see Additional file 2: Table S1). The intake of 11.4 μg Ag NP/kg b.w./d was based on the estimated human dietary intake of silver (70–90 μg/d, i.e. 1.14 μg/kg b.w./d for a 70 kg man) increased by a factor 10 because of the likely underestimation, as already mentioned, and the relatively short exposure duration. Recorded pellet consumption and b.w. allowed to calculate the effective intake that was 60–75 % of the target intake (Additional file 2: Table S1), the highest dose here being below the levels (1.18 to 36 mg/kg b.w./d) used in previous studies in pigs, rats and mice [10, 30, 32, 34].

**Fig. 2:**
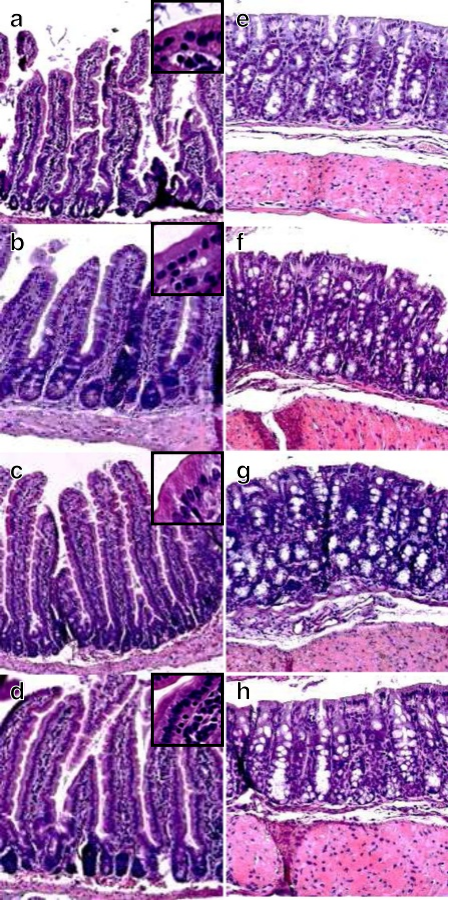
Morphological effects of Ag NP on ileum villi and colonic mucosa. Tissue was collected from mice exposed during 28 d to Ag NP. Sections were stained with hematoxylin and eosin. **a**-**d** Ileum villi and cell coat glycocalyx (inserts) and **e**-**h** colonic mucosa from control mice (**a**, **e**) and mice exposed to 46 (**b**, **f**), 460 (**c**, **g**) and 4600 ppb (**d**, **h**) Ag NP. Magnification 48.7 x, 320 x and 52 x for ileum villi, glycocalyx and colonic mucosa, respectively

No mortality was recorded. B.w. evolution appeared constantly and similarly increasing in all groups, indicating no overt toxicity (Additional file 2: Table S1 and Additional file 3: Figure S1A). Serum C-Reactive Protein (C-RP) level, assessed as an overall evaluation of the possible systemic impact of the treatment, did not show any significant change in Ag NP-treated groups compared to the controls (Additional file 3: Figure S1B). Histological analyses did not reveal intestinal damage or structural alterations: ileal villi were well conserved, goblet cells were unaffected and the glycocalyx integrity was intact in all experimental groups (Fig. 2a–d). Colonic tissue was unaffected by the treatment as shown in Fig. 2e–h. We concluded that Ag NP, at doses used in this study, did not induce overt local (digestive) or systemic toxicity after 28 d. In rats and mice, previous studies showed a decrease of b.w. after gavage exposure to Ag NP and damage to intestinal epithelial cells at doses 5 to 500-fold higher than the highest dose used in the present study [9, 11, 12] while others showed no difference in b.w. [13, 14, 16, 30]. Increase in b.w. were only observed in weaned pigs 14 days after exposure to doses similar to the highest dose used in the present study [10]. Differences in experimental conditions (mainly doses and species, but also modes of administration, NP size and coating and duration of exposure) could explain the discrepancies between studies. However, further investigations with doses relevant for human dietary intake should be conducted with longer exposure durations to better assess the effect of Ag NP intake on the b.w.

### Ag NP disturb the gut microbiota α-diversity

**Fig. 3:**
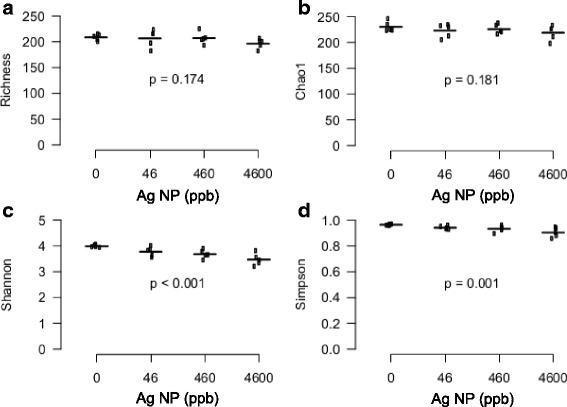
Alpha-diversity of the gut microbiota in mice orally exposed to Ag NP. α-diversity is presented as richness (**a**), Chao1 index (**b**), Shannon index (**c**) and Simpson index (**d**). P-values were obtained with a linear trend test and adjusted with Benjamini-Hochberg method to control FDR (*n* = 5)

After 28 d of exposure to food supplemented with Ag NP, bacterial DNA in mouse feces was analyzed by NGS. After multiple preprocessing steps (see Methods), an OTU table including 285 operational taxonomic units (OTUs) was generated from NGS sequences and α-diversity indexes were calculated. The α-diversity indexes define the richness (number of OTUs) and evenness (relative abundances of OTUs) within a microbial community either qualitatively (Richness and Chao1 indexes) or quantitatively (Shannon and Simpson indexes). The Richness (based on the number of OTUs) and Chao1 (based on the rare OTUs) indexes determine the richness in a community while the Shannon and Simpson indexes determine the richness and evenness of a community [35]. Figure 3a and b indicate that exposure to Ag NP had no significant impact on the richness of the gut microbiota. However, it slightly but significantly decreased the gut microbiota evenness (Fig. 3c and d), a change that is often related to pathological conditions and has been associated with obesity [24], diabetes [32], inflammatory bowel disease [36] and even asthma [29].

### Ag NP dose-dependently disturb gut microbial structure

**Fig. 4:**
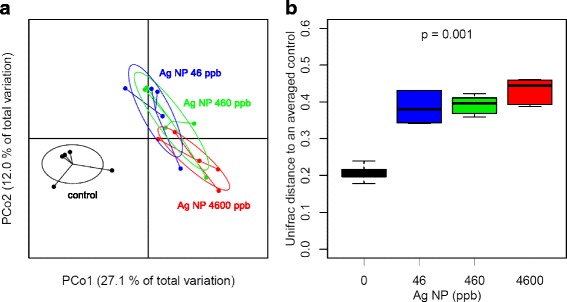
Weighted UniFrac distance analysis. **a** Principal Coordinates Analysis (PCoA) is based on the UniFrac distance matrix generated from all samples of each group of Ag NP dose (0, 46, 460, 4600 ppb). Control samples tend to form a cluster separated from Ag NP treated samples, highlighting the proximity and the similarity of their microbial composition within groups and the significant effect of the treatment. **b** The boxplot of the UniFrac distances shows that distances between treated samples and the averaged control profile increasing with the dose. This dose-dependent effect was confirmed by the permutational multivariate analysis of variance (ADONIS) of the weighted UniFrac distance matrix by assuming a linear trend effect of the Ag NP dose on the microbial structure (*p* < 0.001, *n* = 5)

The diversity between samples (partitioning of diversity or differences among communities, called β-diversity) was analyzed by computing a Generalized UniFrac distance between each sample pair (see Methods). A UniFrac analysis consists in computing a distance metric to compare biological communities and which incorporates information on the relative relatedness of community members by incorporating phylogenetic distances between observed organisms [37]. The principal coordinates analysis (PCoA) of the UniFrac distance matrix enabled to visualize the effect of the Ag NP dose on the microbial structure (Fig. 4a). In addition, the permutational analysis of variance (ADONIS) performed on the UniFrac distance matrix showed a significant linear trend effect of the Ag NP dose on the gut microbial structure, as shown in the boxplot (Fig. 4b).

### Ag NP disturb the gut Firmicutes/Bacteroidetes balance

**Fig. 5:**
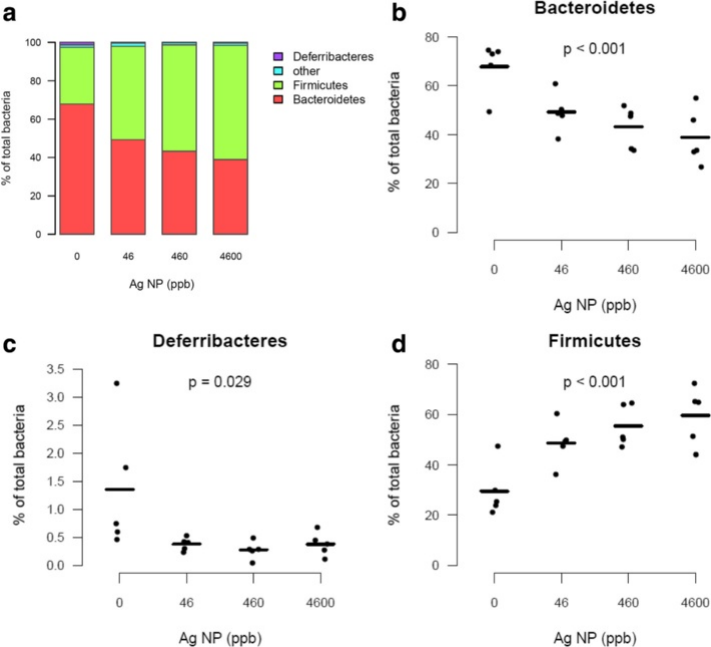
Abundance of bacterial phyla in the gut microbiota after exposure to Ag NP. **a** Relative abundance of the main phyla averaged for each group of Ag NP dose (0, 46, 460, 4600 ppb). **b**-**d** Scatter plots obtained for the most abundant phyla (average relative abundance ≥ 1 % in at least one group). P-values were obtained with a linear trend test and adjusted with Benjamini-Hochberg method to control FDR (*n* = 5)

After taxonomic assignment of the OTUs, the effect of Ag NP was assessed on the abundance of phyla, families, and genera. Phyla in control mice were mainly identified as Firmicutes and Bacteroidetes, which are known to compose approximately 90 % of the mouse and human gut microbiota [38, 39]. Exposure to Ag NP significantly increased the Firmicutes/Bacteroidetes (F/B) ratio in a dose-dependent manner (Fig. 5). Since Firmicutes are mainly Gram-positive bacteria while Bacteroidetes are Gram-negative, this result is consistent with the observations showing that silver ions are more toxic to Gram-negative bacteria that have a thinner wall [17, 40].

In other models, Han also showed that Ag NP ingestion increased the proportion of Firmicutes in the gut microbiota of Drosophila larvae [31] and Das observed a decrease in the proportion of Bacteroidetes after exposure of a human intestinal bacterial mixture to Ag NP [33]. A relative increase of Gram-negative bacteria and a decrease of Firmicutes were observed in rats after 13 weeks of oral gavage with high doses of 10 and 110 nm Ag NP [32]. The latter effects were, however, not dose- and size- dependent. Two other studies showed no effect on the gut microbiota of rats [30] and mice [34] also exposed to high doses of Ag NP. In pigs exposed to Ag NP, the increase of b.w. was associated with a reduction of ileal concentration of coliforms while no difference was detected in the other bacterial groups [10]. These previous studies have been reviewed by Pietroiusti [41] and Frölich [42] in an attempt to elucidate Ag NP mechanisms of action and explain discrepancies between studies. As already mentioned, this could be explained by differences in species, modes of administration (gavage vs diet), doses, NP size and coating, in vitro or in vivo exposure and duration of exposure. While gavage was used in all murine studies [30, 32, 34], oral intake via voluntary consumption in the diet was preferred in the present study because it represents a situation closer to human exposure [43]. Like in bacteria cultured from human stools [33], we observed a relative decrease in Bacteroidetes. Human and mouse gut microbiota are very similar at the phylum level, but not at the genera or species level [41]. Together with the fact that we tested doses and a dosing method more relevant for human dietary intake, this suggests that, at least at the phylum level, our results could be extrapolated to human. The transfer of results from animals to humans could be improved with the use of “humanized” animals by inoculation of human gut microbiota to gnotobiotic mice for instance.

The differences of results between studies could also be related to the techniques used to analyze the microbiota [44]. This includes the type of sample and site of collection, culture-dependent or -independent approach, and techniques to analyze the microbiota (fluorescence in situ hybridization FISH, quantitative (q) PCR or NGS). Many factors can influence the NGS results [44] but it is unlikely that these differences resulted from the choice of the NGS platform (Ion torrent versus Illumina or Roche 454) or the selected hypervariable region in 16 s rRNA gene. It has indeed been shown that different NGS platforms produced concordant relative abundance for most micro-organisms when profiling bacterial community [45] and that using short but well selected region in 16 s rRNA gene produces phylogenetic information similar to that obtained from nearly-full-length 16S rRNA sequencing [46]. However, FISH [10], qPCR [30, 32] and NGS ([31, 33, 34] and the present study) are based on totally different approaches: FISH and qPCR can only detect specific bacteria for which oligonucleotide probes or primers have been selected while NGS can in principle detect any bacteria. Considering studies that analyzed the microbiota by sequencing like in the present study, two of them present similar data at the phylum level [31, 33, 34] and the most recent did not detect any effect of Ag NP [34].

Although baseline mouse microbiota was homogenized before exposure (see Methods), we cannot exclude that our data result from cage effects and stochastic changes over time [47] since all mice from one group were kept in one single cage (one cage/group). However, the observed effects are unlikely due to chance only because alterations of the gut microbiota were significantly dose-dependent.

Data from the present study were also compared to the effects obtained after treatment with antibiotics. In the study where they showed no effect of oral Ag NP on mouse gut microbiota, Wilding observed a significant decrease in α-diversity indexes and an increase of the F/B ratio after exposure to a broad spectrum antibiotic [34]. Other studies also showed a relative reduction in Bacteroidetes with broad spectrum antibiotics [48, 49], which indicates that Ag NP can have similar activities.

Inversion of the gut microbiome F/B ratio has typically been associated with obesity and overweight in mice and in humans [50–53]. Transplantation of gut microbiota from obese animals into the gut of lean mice induced higher fat gain than transplantation of the gut microbiota from lean mice. Thus, one might wonder if Ag NP might promote the development of obesity. We did not observe any impact of Ag NP on mice b.w. over a period of 28 d, and it might be worth considering a longer chronic exposure to Ag NP that might be needed to lead to a (pathological) weight gain.

### Ag NP disturb the abundance of bacterial families and genera

**Fig. 6:**
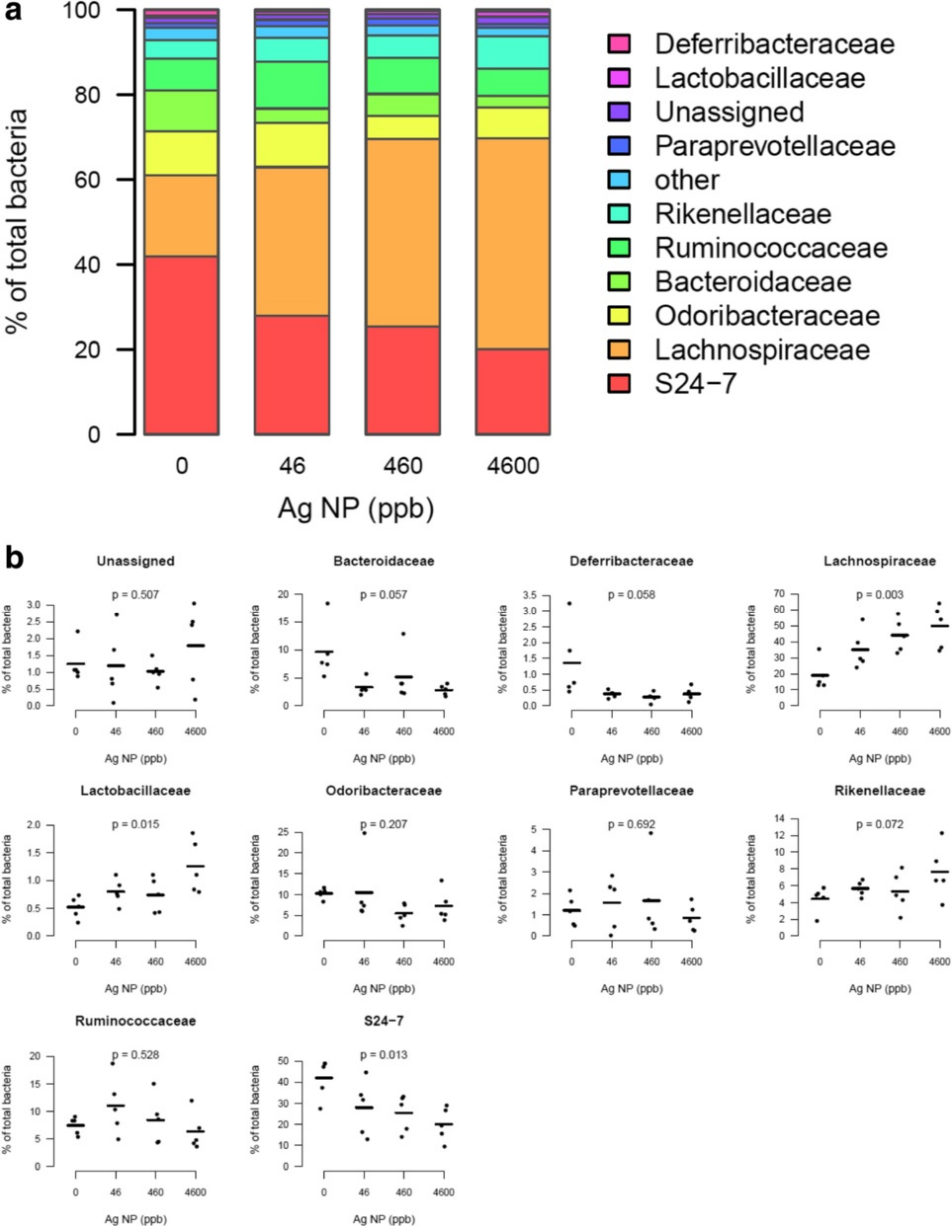
Abundance of bacterial families in the gut microbiota after exposure to Ag NP. **a** Relative abundance of the main families averaged for each group of Ag NP dose (0, 46, 460, 4600 ppb). **b** Scatter plots obtained for the most abundant families (average relative abundance ≥ 1 % in at least one group). P-values were obtained with a linear trend test and adjusted with Benjamini-Hochberg method to control FDR (*n* = 5)

Taxonomic assignment identified 23 families among OTUs. Among all sequences, 99 % were mapped against OTUs with an assigned family (Fig. 6a). The most abundant families included Deferribacteraceae, Lactobacillaceae, Paraprevotellaceae, Rikenellaceae, Ruminococcaceae, Bacteroidaceae, Odoribacteraceae, Lachnospiraceae, and S24 − 7 family. Upon treatment with Ag NP, the decrease in Odoribacteraceae, Bacteroidaceae and S24-7 family and the increase in Lactobacillaceae and Lachnospiraceae accounted for the reduction of Bacteroidetes and increase of Firmicutes, respectively (Fig. 6b). The S24-7 family (unknown genus) has been detected in mouse feces in several other studies, and represents a high proportion of the bacterial population [54–57]. This family is poorly characterized. A very recent study suggested that S24-7 could act as a protective factor against diabetes [58]. In Drosophila larvae, gut Lactobacillaceae were also increased by Ag NP [31].

**Fig. 7:**
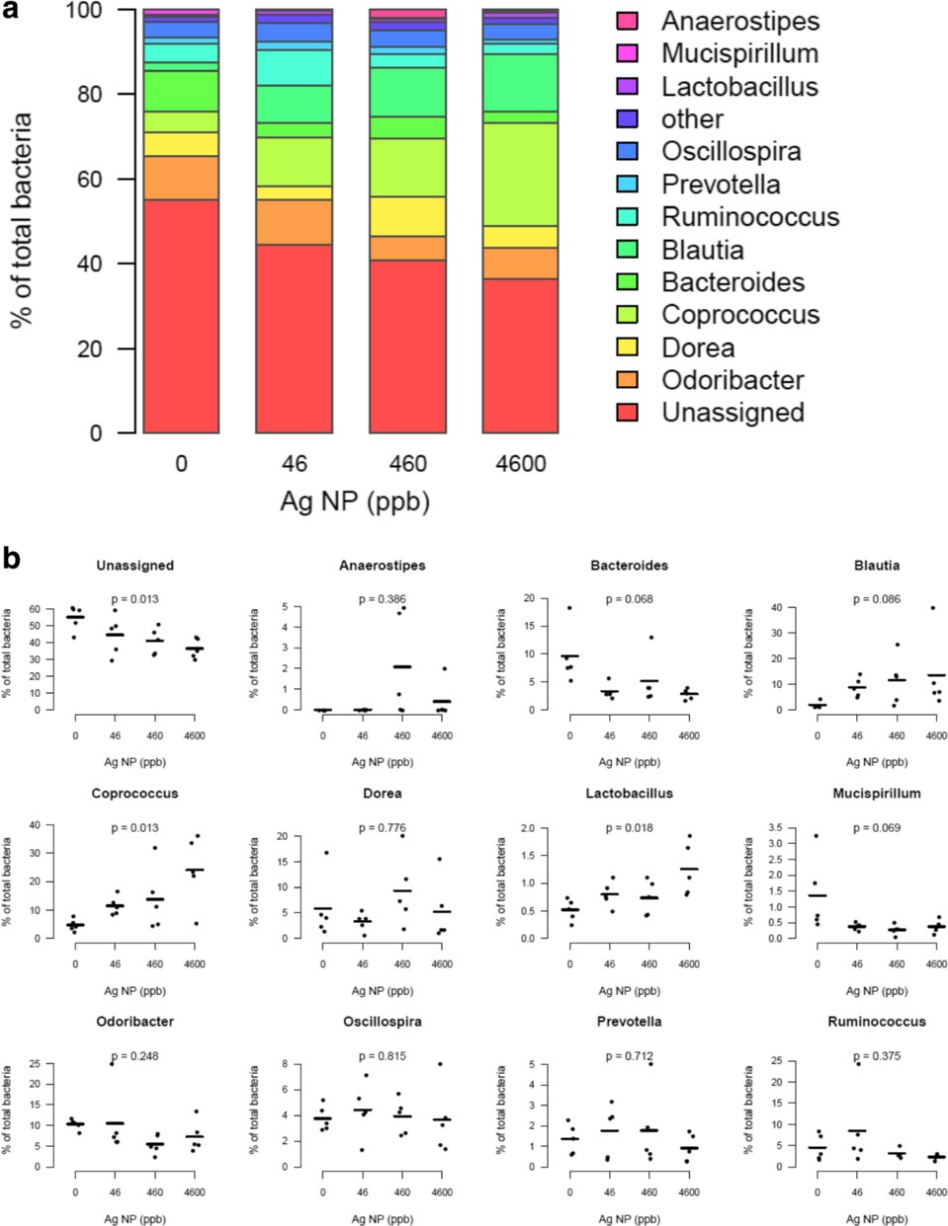
Abundance of bacterial genera in the gut microbiota after exposure to Ag NP. **a** Relative abundance of the main genera averaged for each group of Ag NP dose (0, 46, 460, 4600 ppb). **b** Scatter plots obtained for the most abundant genera (average relative abundance ≥ 1 % in at least one group). P-values were obtained with a linear trend test and adjusted with Benjamini-Hochberg method to control FDR (*n* = 5)

Taxonomic assignment identified 24 genera among OTUs. Among all sequences, 56 % were mapped against OTUs with an assigned genus (Fig. 7a). S24-7 family accounted for most unidentified Bacteroidetes. Relative abundance of Bacteroides genera was reduced by the Ag NP treatment, and Coprococcus, Lactobacillus and Blautia were increased (Fig. 7b). Our data present similarities with several mouse studies showing that high fat diet induces an increase of the Clostridiales order (that includes the family Lachnospiraceae strongly increased in the present study) and a decrease of the Bacteroidales order (that includes Bacteroides genera decreased here) [28, 59]. In humans, Bacteroides are decreased and Coprococcus increased in obese individuals [28, 50]. However, opposite results on Bacteroides were also observed in other studies [28, 60]. Overall, our data show that oral exposure to Ag NP dose-dependently decreases bacterial α-diversity and disturbs taxonomic balance.

### Effect of Ag NP ageing in food

**Fig. 8:**
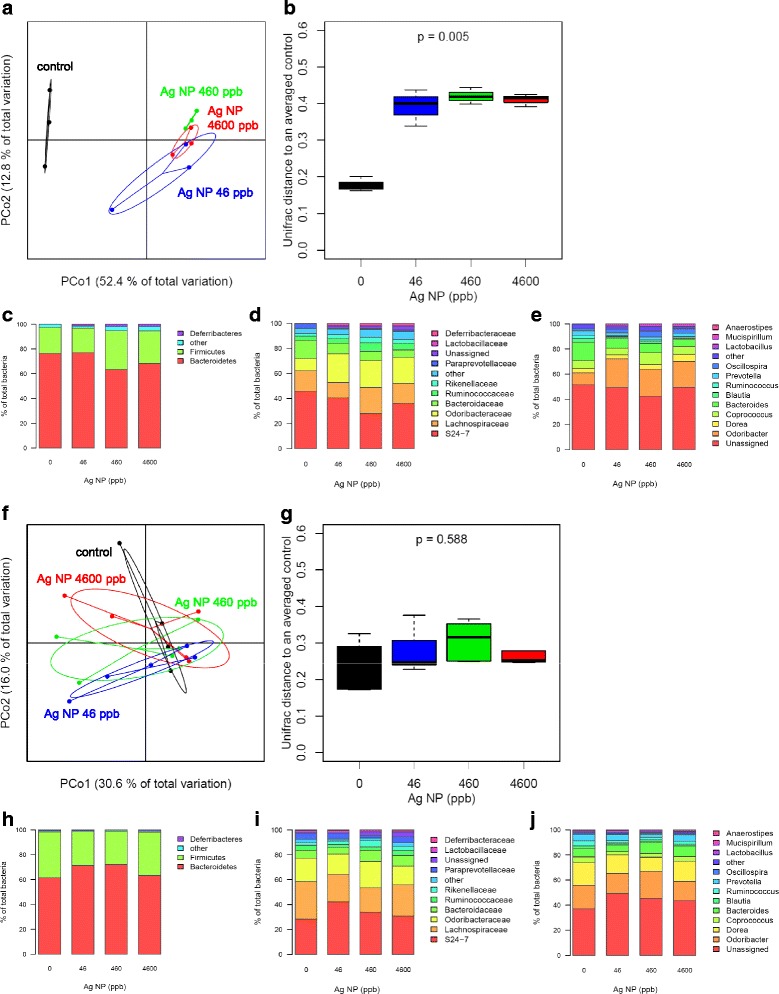
Effect of aged Ag NP supplemented pellets on the gut microbiota. Gut microbiota analysis of mice orally exposed during 28 days to 4-month (A-E, *n* = 3) and 8-month (F-J, *n* = 5) old Ag NP supplemented pellets. **a**, **f** PCoA based on the UniFrac distance matrix, **b**, **g** boxplots of the UniFrac distances, **c**, **h** relative abundance of the main phyla, **d**, **i** families and **e**, **j** genera

Ag NP are known to be highly dynamic in organic environments and food is not necessarily ingested directly after contact with Ag NP. Therefore, we studied the effect of the same batch of Ag NP-supplemented pellets in an experiment repeated under the same conditions 4 and 8 months later, i.e. approximately 4 and 8 months after the incorporation of Ag NP in the pellets. In the second experiment (4 months later), we observed that Ag NP significantly altered gut microbiota (see PCoA in Fig. 8a and b). However, the dose-dependent inversion of F/B balance was less clear with a non-significant decrease of Bacteroidetes (*p* = 0.08) and a non-significant increase of Firmicutes (*p* = 0.11) (Fig. 8c). In the third experiment (8 months after the first experiment), no significant effect of Ag NP was detected and an inversion of F/B balance was not observed (Figs. 8f–j). As the release of Ag ions, related to NP dissolution, is considered so far as the major mechanism of Ag NP toxicity in bacteria [61], we explored whether alteration of Ag NP over time could explain the discrepancies between these 3 experiments.

### Ag sulfidation in aged pellets

**Fig. 9:**
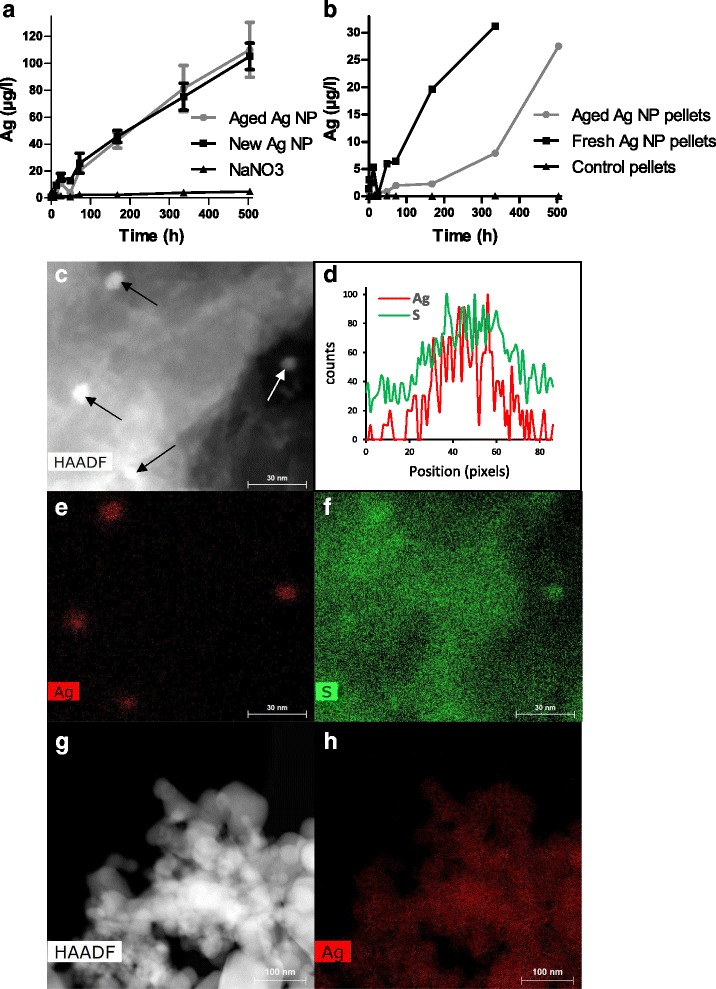
Ag solubilization and sulfidation in Ag NP supplemented pellets. Kinetics of Ag solubilization from **a** aged and new Ag NP and in **b** aged (8 months) and freshly prepared Ag NP-supplemented and control pellets. **c-h** Elemental maps of Ag NP pellets and Ag NP acquired in STEM mode. **c** HAADF image of Ag NP pellets. Fours NP are visible (indicated by arrows). **d** EDX Ag and S profiles, **e** Ag and **f** S maps of Ag NP pellets acquired from EDX analysis. **g** HAADF image and **h** Ag map acquired from EDX analysis of Ag NP alone

We compared the dissolution of newly-acquired Ag NP to that of 8-month old Ag NP conserved in air (not in food pellets). Over a period of 21 d, we did not observe any difference in Ag^+^ release (Fig. 9a). We then assessed the release of Ag ions from freshly prepared (new) and 8-month old (aged) pellets supplemented with Ag NP. As shown in Fig. 9b, aged pellets released Ag^+^ less rapidly than new ones. Thus Ag ions were less bioaccessible in pellets used in the 3^rd^ experiment than in those used in the 1^rst^ experiment.

Sulfidation of Ag NP is known to occur in the environment, even if NP are coated with PVP, and to reduce dissolution rate and modify surface charge [62]. This sulfidation was shown to decrease Ag NP toxicity to bacteria, fish, nematodes and plants [61, 63]. We then studied Ag sulfidation in aged pellets by scanning transmission electron microscopy (STEM) and energy dispersive X-ray analysis (EDX). Ag NP observed in 8-month old supplemented pellets showed the concomitant presence of Ag and S (Fig. 9c–f). Ag NP that had not been mixed with pellets did not show any sulfur (Fig. 9g and h). Thus, Ag NP solubilization and alteration of the gut microbiota decreased along with Ag NP sulfidation and time spent in contact with food, suggesting that Ag^+^ release is probably an important component of the Ag NP toxicity for the gut microbiota, although we cannot exclude that effects could also be mediated by the nanoparticle form. Sulfidation is a major transformation process for Ag NP in contact with organic materials containing thiols, sulfur-containing amino acids or proteins. Sulfidation of Ag NP has been reported in wastewater treatment systems [64], soils [65] or cell culture media [66]. To the best of our knowledge, this study is the first to report sulfidation of Ag NP in food. How this observation can be translated to human exposure scenario will vary according to food composition and duration of contact with Ag NP. Moreover, although most previous studies with Ag NP were performed by gavage (no incorporation of Ag NP to food), it cannot be excluded that, in some studies, Ag NP samples were inactivated by sulfidation and that this could explain inconsistent results. We, therefore, recommend that the sulfidation state of Ag NP samples be systematically documented in future studies.

## Conclusions

Our data indicate that oral exposure to Ag NP at doses relevant for human dietary intake can induce microbial alterations in the gut. It suggests that Ag NP aging in food, and more specifically sulfidation, can reduce the effects on the microbiota and that the potential risk of microbial alteration from human exposure to Ag NP in foods or food containers is likely to relate to the ability of Ag NP to release silver ions. It appears, therefore, necessary to assess the degree, rate and duration of Ag^+^ release over time and under likely exposure conditions for predicting the potential of new Ag NP-containing products to alter microbial communities.

This study also highlights that Ag NP can induce gut microbial alterations, specifically an increased F/B ratio, similar to that described in association with obesity and inflammatory diseases, as well as with antibiotic administration. However, the 28-day protocol applied in the present study did not detect systemic impacts or increased weight gain associated to microbial alterations induced by Ag NP. Further investigations should be conducted with longer exposure durations and using regularly renewed Ag NP pellets to better mimic human exposure scenarios.

## Methods

### Nanoparticle characterization

Polyvinylpyrrolidone (PVP)-coated Ag NP particles were obtained from Sigma-Aldrich (Silver 576832-5G, Vial # MKBN3581V, MO, USA). NP purity was 99.5 % and PVP coating represented 0.2 %, as stated by the provider. NP surface area, porosity and size were characterized by N_2_ physisorption and TEM, respectively (see Additional file 1).

### Preparation of pellets supplemented in Ag NP

Mouse food, i.e. lab pellets from Carfil (Rats & Mice Maintenance, Oud-Turnhout, Belgium), was supplemented with 0, 46, 460 or 4600 ppb Ag NP by the provider using appropriate aliquots of Ag NP incorporated during the preparation of the pellets to reach the target NP concentrations. Food bags were γ-irradiated and stored at room temperature, in dark and dry conditions.

### Solubilization of Ag from Ag NP and pellets

Aged (8 months) and newly prepared pellets supplemented with Ag NP (46 ppb), pristine (control) pellets, aged (8 months) and new Ag NP were suspended in 10 ml NaNO_3_ 0.01 M (pH7), incubated under slow rotation at room temperature during 4 weeks. Aliquots were collected at different time points, centrifuged at 12000 g and supernatants were analyzed by inductively coupled plasma mass spectrometry (ICP-MS) for Ag content.

### Ag and S co-localization in Ag NP-supplemented pellets

Pellet samples first ground into fine powder or Ag NP were diluted in pure EtOH. Three μL of solution was deposed on a carbon grid fil (Ted Pella 1824) first prepared to avoid agglomeration using glow discharge plasma (Ar/O_2_, 25 W, 20 s). TEM carbon films were then observed on a Tecnai Osiris TEM commercialized by FEI. Images were recorded in STEM mode. Images were recorded using a HAADF detector (High Angle Annular Dark Field). This detector gives a contrast proportional to the mean Z of the element and the thickness of the sample. EDX acquisition was carried out in STEM mode (a probe size of approximately 5 Angström, probe current of 0.4 nA) and the 4 detectors of the system. The solid angle is thus close to 1 srad. Spectra images were recorded for each sample and element identification was carried out on a representative area of the sample. Acquisition time was 10 min.

### Animals and experimental design

Experiments were set up according to OECD guideline 407 for testing of chemicals (“Repeated dose 28-d oral study in rodents”). Three month old C57BL/6 female mice, locally bred under SPF-like conditions (Animalerie Centrale, Université catholique de Louvain, Brussels, Belgium) in a controlled environment (22 °C, 55 % relative humidity, 16-h light/8-h dark cycle, with acidified water and food *ad libitum*) were housed in autoclaved air-filtered polycarbonate cages with conventional sawdust (Carfil). Litters were mixed every day for one week before exposure to homogenize the baseline gut microbiota [67]. Mice were then randomly assigned to experimental groups (one cage/dose group) and fed with control or Ag NP-supplemented pellets, i.e. orally exposed to food supplemented with 0, 46, 460 or 4600 ppb Ag NP (μg Ag NP/kg pellet), during 28 d. A single experimenter took care of the animals during the experimental period. Control pellets were not supplemented with free PVP since this might not represent PVP coated on particles and since PVP is not expected to have any effect on mammalian cells, nor on bacteria [68, 69].

Mice were weighed prior to and twice a week during the experimental period and food consumption was recorded per cage to calculate the actual Ag NP daily intake. After 28 d exposure, mice were euthanized by an intra-muscular injection of 60 mg of pentobarbital and intra-cardiac blood was collected. The 3 distal centimeters of the ileum, the caecum, and the 3 proximal centimeters of the colon were excised. Intestines were opened longitudinally and their content was gently removed by scraping. Those 3 feces samples were collected and pooled per mouse, and stored at −20 °C until DNA extraction. The outer side of the gut tissues was applied against blotting paper which was then submerged in 10 % formaldehyde, inner side downwards, allowing intestinal villi to stretch with gravity. Hematoxylin-eosin (H&E) staining was performed on 5 μm thick intestinal sections.

All animal experiments were performed in accordance with local and institutional ethical guidelines.

### Serum analyses

Serum C-Reactive Protein (C-RP) was measured by a magnetic Luminex screening assay according to manufacturer’s instructions (R&D Systems, Minneapolis, USA) on a Bio-Plex 200 analyzer (Bio-Rad, Hercules, California, USA).

### DNA extraction and 16 s rRNA gene sequencing

Feces DNA was extracted with a QIAamp DNA Stool Mini kit according to manufacturer’s guidelines (Qiagen, Hilden, Germany), except for DNA elution with sterilized PCR-grade water (Merck, Darmstadt, Germany). DNA concentrations were calculated on a Qubit Fluorometer (Invitrogen, Gent, Belgium) with a Qubit dsDNA HS Assay kit. DNA samples were stored at −20 °C until NGS analysis at MR DNA lab (Shallowater, USA).

The gut microbial populations were identified and quantified on an Ion Torrent Personal Genome Machine (PGM) (Life technologies, USA). Specific primers of the 16S rRNA gene V4 variable region (forward GTGCCAGCMGCCGCGGTAA and reverse GGACTACHVGGGTWTCTAAT [70]) were used in a PCR with the HotStarTaq Plus Master Mix Kit (Qiagen) under the following conditions: 94 °C 3 min, (94 °C 30 s, 53 °C 40 s, 72 °C 1 min) x28, 72 °C 5 min. PCR was made in a single-step, meaning that a sample-specific barcode and a global primer was added to each amplicon which were all sequenced in a single run on an Ion Torrent PGM following the manufacturer’s guidelines.

### NGS data analysis

Raw sequences generated by the NGS experiments were processed using USEARCH v8.0.1623 (Tiburon, USA) for linux in order to perform a de-novo OTU clustering analysis [71, 72]. A quality filtering step was applied in order to keep sequences with an expected number of errors <1.5 and a length >150 nt. After the dereplication and the sorting of the remaining sequences, the latter were clustered into representative OTUs using a radius percentage of 3.0. The resulting OTUs were then scanned to detect and to remove the chimera. These steps were performed on a pool of sequences generated by all samples from the 3 experiments (0, 4, and 8 months), as recommended in the USEARCH manual in order to make the results of each experiment comparable. The quality-filtered sequences from all samples of each experiment were mapped against the non-chimeric OTUs (*n* = 343) and one OTU table was therefore generated for each experiment.

The taxonomic assignment of each OTU was carried out using a local BLAST against the Greengenes database version 13.5 [73]. The Greengenes database was divided into 4 sub-databases according to the level of taxonomic definition (genus, family, order, phylum). The taxonomic assignment of each OTU was based on a blast hit at the species, genus, family or order level if a > 97 %, > 95 %, > 90 % or > 85 % identity was found in the corresponding databases, respectively.

The OTU tables and the OTU taxonomic assignments were the starting point of the downstream analyses which were performed using the R software (version 3.1.2., Vienna, Austria). A second level of quality-filtering based on OTU abundance was carried out to discard those OTUs with a number of sequences <0.005 % of the total number of sequences, as recommended before [74]. This second level of quality-filtering resulted into an OTU table including 285 OTUs. After rarefaction, the α-diversity indexes (Richness, Chao1, Simpson and Shannon) were computed for each sample with the phyloseq R package [75] while the β-diversity, which involves the assessment of dissimilarity between the composition of communities by taking into account the evolutionary relationship between sequences, analyses were carried out by computing weighted UniFrac distances with the GUnifrac R package [76]. The UniFrac analysis was based on a phylogenetic tree of the OTUs which was obtained with the UPGMA algorithm after OTU sequence alignment with MUSCLE function [77] from the muscle R package. The α parameter of the weighted UniFrac distance analysis was set to 0.5 in order to avoid that distances be dominated by highly abundant lineages. The weighted UniFrac distance matrix was visualized using a principal coordinates analysis (PCoA) with the cmdscale function from the GUnifrac R package. In addition, the ADONIS function of the same package was used to perform a permutational multivariate analysis of variance of the weighted UniFrac distance matrix in order to detect the association between the Ag NP dose and the β-diversity.

The association between Ag NP dose and each outcome (including α- and β-diversities as well as phyla, family and genera relative abundance) were assessed via linear trend effects which were computed using linear regression model by assuming equally spaced groups (0, 46, 460, and 4600 ppb). When the effect of the Ag NP dose was assessed on multiple outcomes (e.g. 11 family relative abundances or 4 α-diversity measures), p-values were corrected with the Benjamini-Hochberg method in order to control the False Discovery Rate (FDR) [78], and the significance threshold for the adjusted p-values was set at 0.05.

## Abbreviations

b.w., body weight; C-RP, C-Reactive Protein; EDX, energy dispersive X-ray analysis; F/B, Firmicutes/Bacteroidetes; FDR, False Discovery Rate; H&E, hematoxylin-eosin; HAADF, High Angle Annular Dark Field; ICP-MS, inductively coupled plasma mass spectrometry; NGS, Next Generation Sequencing; NM, nanomaterials; NOAEL, no observed adverse effect level; NP, nanoparticles; OTU, operational taxonomic unit; PCoA, principal coordinates analysis; PGM, Personal Genome Machine; ppb, part per billion (μg/kg); PVP, polyvinylpyrrolidone; TEM, transmission electron microscopy

## Additional files

Additional file 1: Methods. (PDF 48 kb)

Additional file 2: Table S1. Oral exposure to Ag NP and mouse final b.w. (PDF 36 kb)

Additional file 3: Figure S1. B.w. and blood C-RP responses in mice orally exposed to Ag NP during 28 d. C57BL/6 female mice were orally exposed to food supplemented with 0, 46, 460 or 4600 ppb nAg during 28 d. (A) B.w. were measured on d 0 prior to first exposure and twice a week during the experimental period. (B) C-RP was quantified in serum by Luminex. Graphs represent means ± SEM (*n* = 3-5). (PDF 38 kb)

## References

[1] Chaudhry Q, Scotter M, Blackburn J, Ross B, Boxall A, Castle L (2008). Applications and implications of nanotechnologies for the food sector. Food Addit Contam Part A Chem Anal Control Expo Risk Assess.

[2] Srinivas PR, Philbert M, Vu TQ, Huang Q, Kokini JL, Saltos E (2010). Nanotechnology research: applications in nutritional sciences. J Nutr.

[3] Hwang M, Lee EJ, Kweon SY, Park MS, Jeong JY, Um JH (2012). Risk assessment principle for engineered nanotechnology in food and drug. Toxicol Res.

[4] Consumer Products Inventory, http://www.nanotechproject.org/cpi/. Accessed 29 Jun 2016.

[5] Wijnhoven SWP, Peijnenburg WJGM, Herberts CA, Hagens WI, Oomen AG, Heugens EHW (2009). Nano-silver – a review of available data and knowledge gaps in human and environmental risk assessment. Nanotoxicology.

[6] Bohmert L, Niemann B, Lichtenstein D, Juling S, Lampen A (2015). Molecular mechanism of silver nanoparticles in human intestinal cells. Nanotoxicology.

[7] van der Zande M, Vandebriel RJ, Van DE, Kramer E, Herrera RZ, Serrano-Rojero CS (2012). Distribution, elimination, and toxicity of silver nanoparticles and silver ions in rats after 28-day oral exposure. ACS Nano.

[8] Lee JH, Kim YS, Song KS, Ryu HR, Sung JH, Park JD (2013). Biopersistence of silver nanoparticles in tissues from Sprague–Dawley rats. Part Fibre Toxicol.

[9] Ebabe ER, Gaillet S, Vide J, Romain C, Lauret C, Rugani N (2013). Dietary exposure to silver nanoparticles in Sprague–Dawley rats: effects on oxidative stress and inflammation. Food Chem Toxicol.

[10] Fondevila M, Herrer R, Casallas MC, Abecia L, Ducha JJ (2009). Silver nanoparticles as a potential antimicrobial additive for weaned pigs. Anim Feed Sci Technol.

[11] Kim YS, Song MY, Park JD, Song KS, Ryu HR, Chung YH (2010). Subchronic oral toxicity of silver nanoparticles. Part Fibre Toxicol.

[12] Shahare B, Yashpal M (2013). Toxic effects of repeated oral exposure of silver nanoparticles on small intestine mucosa of mice. Toxicol Mech Methods.

[13] Kim YS, Kim JS, Cho HS, Rha DS, Kim JM, Park JD (2008). Twenty-eight-day oral toxicity, genotoxicity, and gender-related tissue distribution of silver nanoparticles in Sprague–Dawley rats. Inhal Toxicol.

[14] Park K (2013). Toxicokinetic differences and toxicities of silver nanoparticles and silver ions in rats after single oral administration. J Toxicol Environ Health A.

[15] Bergin IL, Wilding LA, Morishita M, Walacavage K, Ault AP, Axson JL (2016). Effects of particle size and coating on toxicologic parameters, fecal elimination kinetics and tissue distribution of acutely ingested silver nanoparticles in a mouse model. Nanotoxicology.

[16] Park EJ, Bae E, Yi J, Kim Y, Choi K, Lee SH (2010). Repeated-dose toxicity and inflammatory responses in mice by oral administration of silver nanoparticles. Environ Toxicol Pharmacol.

[17] Volker C, Oetken M, Oehlmann J (2013). The biological effects and possible modes of action of nanosilver. Rev Environ Contam Toxicol.

[18] Aitken JD, Gewirtz AT (2013). Gut microbiota in 2012: toward understanding and manipulating the gut microbiota. Nat Rev Gastroenterol Hepatol.

[19] Larsson E, Tremaroli V, Lee YS, Koren O, Nookaew I, Fricker A (2012). Analysis of gut microbial regulation of host gene expression along the length of the gut and regulation of gut microbial ecology through MyD88. Gut.

[20] Maurice CF, Haiser HJ, Turnbaugh PJ (2013). Xenobiotics shape the physiology and gene expression of the active human gut microbiome. Cell.

[21] Flint HJ, Scott KP, Duncan SH, Louis P, Forano E (2012). Microbial degradation of complex carbohydrates in the gut. Gut Microbes.

[22] Kostic AD, Howitt MR, Garrett WS (2013). Exploring host-microbiota interactions in animal models and humans. Genes Dev.

[23] Yatsunenko T, Rey FE, Manary MJ, Trehan I, Dominguez-Bello MG, Contreras M (2012). Human gut microbiome viewed across age and geography. Nature.

[24] Thaiss CA, Zeevi D, Levy M, Zilberman-Schapira G, Suez J, Tengeler AC (2014). Transkingdom control of microbiota diurnal oscillations promotes metabolic homeostasis. Cell.

[25] Le Chatelier E, Nielsen T, Qin J, Prifti E, Hildebrand F, Falony G (2013). Richness of human gut microbiome correlates with metabolic markers. Nature.

[26] Suez J, Korem T, Zeevi D, Zilberman-Schapira G, Thaiss CA, Maza O (2014). Artificial sweeteners induce glucose intolerance by altering the gut microbiota. Nature.

[27] Chassaing B, Koren O, Goodrich JK, Poole AC, Srinivasan S, Ley RE (2015). Dietary emulsifiers impact the mouse gut microbiota promoting colitis and metabolic syndrome. Nature.

[28] Delzenne NM, Cani PD (2011). Interaction between obesity and the gut microbiota: relevance in nutrition. Annu Rev Nutr.

[29] Huang YJ, Boushey HA (2015). The microbiome in asthma. J Allergy Clin Immunol.

[30] Hadrup N, Loeschner K, Bergstrom A, Wilcks A, Gao X, Vogel U (2012). Subacute oral toxicity investigation of nanoparticulate and ionic silver in rats. Arch Toxicol.

[31] Han X, Geller B, Moniz K, Das P, Chippindale AK, Walker VK (2014). Monitoring the developmental impact of copper and silver nanoparticle exposure in Drosophila and their microbiomes. Sci Total Environ.

[32] Williams K, Milner J, Boudreau MD, Gokulan K, Cerniglia CE, Khare S (2015). Effects of subchronic exposure of silver nanoparticles on intestinal microbiota and gut-associated immune responses in the ileum of Sprague–Dawley rats. Nanotoxicology.

[33] Das P, McDonald JAK, Petrof EO, Allen-Vercoe E, Walker VK (2014). Nanosilver-mediated change in human intestinal microbiota. Journal of Nanomedicine & Nanotechnology.

[34] Wilding LA, Bassis CM, Walacavage K, Hashway S, Leroueil PR, Morishita M (2016). Repeated dose (28-day) administration of silver nanoparticles of varied size and coating does not significantly alter the indigenous murine gut microbiome. Nanotoxicology.

[35] Lozupone CA, Knight R (2008). Species divergence and the measurement of microbial diversity. FEMS Microbiol Rev.

[36] Kim A (2015). Dysbiosis: a review highlighting obesity and inflammatory bowel disease. J Clin Gastroenterol.

[37] Lozupone C, Hamady M, Knight R (2006). UniFrac--an online tool for comparing microbial community diversity in a phylogenetic context. BMC Bioinformatics.

[38] Hur KY, Lee MS (2015). Gut microbiota and metabolic disorders. Diabetes Metab J.

[39] Schippa S, Conte MP (2014). Dysbiotic events in gut microbiota: impact on human health. Nutrients.

[40] Jung WK, Koo HC, Kim KW, Shin S, Kim SH, Park YH (2008). Antibacterial activity and mechanism of action of the silver ion in Staphylococcus aureus and Escherichia coli. Appl Environ Microbiol.

[41] Pietroiusti A, Magrini A, Campagnolo L (2016). New frontiers in nanotoxicology: gut microbiota/microbiome-mediated effects of engineered nanomaterials. Toxicol Appl Pharmacol.

[42] Frohlich EE, Frohlich E (2016). Cytotoxicity of Nanoparticles Contained in Food on Intestinal Cells and the Gut Microbiota. Int J Mol Sci..

[43] Vandenberg LN, Welshons WV, Vom Saal FS, Toutain PL, Myers JP (2014). Should oral gavage be abandoned in toxicity testing of endocrine disruptors?. Environ Health.

[44] Goodrich JK, Di Rienzi SC, Poole AC, Koren O, Walters WA, Caporaso JG (2014). Conducting a microbiome study. Cell.

[45] Salipante SJ, Kawashima T, Rosenthal C, Hoogestraat DR, Cummings LA, Sengupta DJ (2014). Performance comparison of Illumina and ion torrent next-generation sequencing platforms for 16S rRNA-based bacterial community profiling. Appl Environ Microbiol.

[46] Jeraldo P, Chia N, Goldenfeld N (2011). On the suitability of short reads of 16S rRNA for phylogeny-based analyses in environmental surveys. Environ Microbiol.

[47] McCafferty J, Muhlbauer M, Gharaibeh RZ, Arthur JC, Perez-Chanona E, Sha W (2013). Stochastic changes over time and not founder effects drive cage effects in microbial community assembly in a mouse model. ISME J.

[48] Antonopoulos DA, Huse SM, Morrison HG, Schmidt TM, Sogin ML, Young VB (2009). Reproducible community dynamics of the gastrointestinal microbiota following antibiotic perturbation. Infect Immun.

[49] Bassis CM, Theriot CM, Young VB (2014). Alteration of the murine gastrointestinal microbiota by tigecycline leads to increased susceptibility to Clostridium difficile infection. Antimicrob Agents Chemother.

[50] Kasai C, Sugimoto K, Moritani I, Tanaka J, Oya Y, Inoue H (2015). Comparison of the gut microbiota composition between obese and non-obese individuals in a Japanese population, as analyzed by terminal restriction fragment length polymorphism and next-generation sequencing. BMC Gastroenterol.

[51] Musso G, Gambino R, Cassader M (2010). Obesity, diabetes, and gut microbiota: the hygiene hypothesis expanded?. Diabetes Care.

[52] Mathur R, Barlow GM (2015). Obesity and the microbiome. Expert Rev Gastroenterol Hepatol.

[53] Lopez-Cepero AA, Palacios C (2015). Association of the intestinal microbiota and obesity. P R Health Sci J.

[54] Sands SA, Tsau S, Yankee TM, Parker BL, Ericsson AC, LeVine SM (2014). The effect of omeprazole on the development of experimental autoimmune encephalomyelitis in C57BL/6 J and SJL/J mice. BMC Res Notes.

[55] Evans CC, LePard KJ, Kwak JW, Stancukas MC, Laskowski S, Dougherty J (2014). Exercise prevents weight gain and alters the gut microbiota in a mouse model of high fat diet-induced obesity. PLoS One.

[56] Serino M, Luche E, Gres S, Baylac A, Berge M, Cenac C (2012). Metabolic adaptation to a high-fat diet is associated with a change in the gut microbiota. Gut.

[57] Rooks MG, Veiga P, Wardwell-Scott LH, Tickle T, Segata N, Michaud M (2014). Gut microbiome composition and function in experimental colitis during active disease and treatment-induced remission. ISME J.

[58] Krych L, Nielsen DS, Hansen AK, Hansen CH (2015). Gut microbial markers are associated with diabetes onset, regulatory imbalance, and IFN-gamma level in NOD mice. Gut Microbes.

[59] Cani PD, Bibiloni R, Knauf C, Waget A, Neyrinck AM, Delzenne NM (2008). Changes in gut microbiota control metabolic endotoxemia-induced inflammation in high-fat diet-induced obesity and diabetes in mice. Diabetes.

[60] Everard A, Lazarevic V, Derrien M, Girard M, Muccioli GG, Neyrinck AM (2011). Responses of gut microbiota and glucose and lipid metabolism to prebiotics in genetic obese and diet-induced leptin-resistant mice. Diabetes.

[61] Levard C, Hotze EM, Colman BP, Dale AL, Truong L, Yang XY (2013). Sulfidation of silver nanoparticles: natural antidote to their toxicity. Environ Sci Technol.

[62] Levard C, Reinsch BC, Michel FM, Oumahi C, Lowry GV, Brown GE (2011). Sulfidation processes of PVP-coated silver nanoparticles in aqueous solution: impact on dissolution rate. Environ Sci Technol.

[63] Reinsch BC, Levard C, Li Z, Ma R, Wise A, Gregory KB (2012). Sulfidation of silver nanoparticles decreases Escherichia coli growth inhibition. Environ Sci Technol.

[64] Doolette CL, McLaughlin MJ, Kirby JK, Batstone DJ, Harris HH, Ge H (2013). Transformation of PVP coated silver nanoparticles in a simulated wastewater treatment process and the effect on microbial communities. Chem Cent J.

[65] Rick VA, Tappero R, Arai Y (2014). Residence time effects on phase transformation of nanosilver in reduced soils. Environ Sci Pollut Res Int.

[66] Chen S, Theodorou IG, Goode AE, Gow A, Schwander S, Zhang JJ (2013). High-resolution analytical electron microscopy reveals cell culture media-induced changes to the chemistry of silver nanowires. Environ Sci Technol.

[67] Ma BW, Bokulich NA, Castillo PA, Kananurak A, Underwood MA, Mills DA (2012). Routine habitat change: a source of unrecognized transient alteration of intestinal microbiota in laboratory mice. PLoS One.

[68] Burkowska-But A, Sionkowski G, Walczak M (2014). Influence of stabilizers on the antimicrobial properties of silver nanoparticles introduced into natural water. J Environ Sci (China).

[69] Silva T, Pokhrel LR, Dubey B, Tolaymat TM, Maier KJ, Liu X (2014). Particle size, surface charge and concentration dependent ecotoxicity of three organo-coated silver nanoparticles: comparison between general linear model-predicted and observed toxicity. Sci Total Environ.

[70] Caporaso JG, Lauber CL, Walters WA, Berg-Lyons D, Lozupone CA, Turnbaugh PJ (2011). Global patterns of 16S rRNA diversity at a depth of millions of sequences per sample. Proc Natl Acad Sci U S A.

[71] Edgar RC (2010). Search and clustering orders of magnitude faster than BLAST. Bioinformatics.

[72] Edgar RC (2013). UPARSE: highly accurate OTU sequences from microbial amplicon reads. Nat Methods.

[73] DeSantis TZ, Hugenholtz P, Larsen N, Rojas M, Brodie EL, Keller K (2006). Greengenes, a chimera-checked 16S rRNA gene database and workbench compatible with ARB. Appl Environ Microbiol.

[74] Navas-Molina JA, Peralta-Sanchez JM, Gonzalez A, McMurdie PJ, Vazquez-Baeza Y, Xu Z (2013). Advancing our understanding of the human microbiome using QIIME. Methods Enzymol.

[75] McMurdie PJ, Holmes S (2013). phyloseq: an R package for reproducible interactive analysis and graphics of microbiome census data. PLoS One.

[76] Chen J, Bittinger K, Charlson ES, Hoffmann C, Lewis J, Wu GD (2012). Associating microbiome composition with environmental covariates using generalized UniFrac distances. Bioinformatics.

[77] Edgar RC (2004). MUSCLE: multiple sequence alignment with high accuracy and high throughput. Nucleic Acids Res.

[78] Benjamini YHY (1995). Controlling the False Discovery Rate: a practical and powerful approach to multiple testing. J Royal Stat Soc Ser B.

