# Supplementary material for: Dietary silver nanoparticles can disturb the gut microbiota in mice
Source: Part Fibre Toxicol. 2016 Jul 8;13:38. doi: 10.1186/s12989-016-0149-1 (PMC4939013; doi:10.1186/s12989-016-0149-1)
Supplement: Supplementary file 1 — Methods. (PDF 48 kb) [file 12989_2016_149_MOESM1_ESM.pdf]

Additional file 1: methods

Methods

Nanoparticle characterization

For determination of NP size by TEM, NP were brought in double distilled water at a concentration of 20 mg/ml by weighing 0.2 g of powder and adding 10 ml of milli-Q water and then sonicated for 30 sec using a Vibracell™ 75041 ultrasonifier (750 W, 20 kHz, Fisher Bioblock Scientific, Aalst, Belgium) equipped with a 13 mm horn (CV33) at 40 % as described in [1]. Samples were cooled with water at room temperature. This setup resulted in an added energy of 1 kJ. The TEM specimen was prepared using the grid on drop method by bringing 15 µl of the diluted dispersion on pioloform- and carbon-coated, 400 mesh copper grids (Agar Scientific, Essex, England) that were pretreated with 1% Alcian blue (Fluka, Buchs, Switzerland) to increase hydrophilicity [2]. The specimen was imaged in BF mode using a Tecnai G2 Spirit TEM (FEI, Eindhoven, The Netherlands) with Biotwin lens configuration operating at 120 kV mounted with a condenser aperture of 100 µm and an objective aperture of 150 µm. Digital micrographs were recorded at magnification of 18500 (0.6 nm/pixel) and taken systematically random at predefined widely separated positions. A bottom-mounted 4x4 K Eagle CCD-camera was used to record the micrographs. Micrographs were analyzed using ImageJ (National Institute of Health, Rockville Pike, USA).

Textural properties of the sample (specific surface area, presence of pores, and if present pore size) were characterized in collaboration with Pr. E. Gaigneaux (Institute of Condensed Matter and Nanosciences, Université catholique de Louvain, Belgium) through nitrogen adsorption–desorption experiments (N_2 physisorption) with a Micromeritics Tristar 3000 apparatus. Before analysis, the sample (approximatively 150 mg) was degassed overnight under vacuum (0.67 Pa) at 150 °C. The measurements were performed at -196 °C and with relative pressures of dinitrogen in the range of 0.01-1.00 (P/P°). The specific surface area was calculated from the adsorption isotherm in the P/P° range of 0.05-0.30 using the BET method, and denoted afterwards SBET. The adsorption isotherm

presents a typical type-II shape. Namely, there is a very small N_2 uptake at low pressure (at P/P° lower than 10^{-5}), which ascertains that the isotherm is not of the type-I typical of microporous samples. Also, there is no N2 uptake at intermediate partial pressures (which would indicate the occurrence of a capillary condensation), which ascertains that the isotherm is not of type-IV which would reflect of the presence of mesopores. The absence of hysteresis between the adsorption and desorption curves additionally argues in this sense. Finally, the asymptotic increase of the isotherm when coming close to the saturation pressure indicates that the adsorption proceeds in a system without sterical constraints allowing N_2 to adsorb in a huge number of layers at the surface of the sample, i.e. not in a porous system. These observations indicate that the sample is made of massive dense particles, neither presenting intraparticular pores inside them, nor developing any significant interparticular pores. This conclusion is in good agreement with the small SBET value found for the sample (5.57 m^2/g) which is typical of dense non porous samples.

References

1. De Temmerman PJ, Van DE, Verleysen E, Van der Stede Y, Francisco MA, Mast J. Quantitative characterization of agglomerates and aggregates of pyrogenic and precipitated amorphous silica nanomaterials by transmission electron microscopy**.** J Nanobiotechnology 2012; 10**:** 24.
2. Mast J, Demeestere L. Electron tomography of negatively stained complex viruses: application in their diagnosis**.** Diagn Pathol 2009; 4**:** 5.
