# Supplementary material for: Dietary silver nanoparticles can disturb the gut microbiota in mice
Source: Part Fibre Toxicol. 2016 Jul 8;13:38. doi: 10.1186/s12989-016-0149-1 (PMC4939013; doi:10.1186/s12989-016-0149-1)
Supplement: Supplementary file 2 — Oral exposure to Ag NP and mouse final b.w. (PDF 36 kb) [file 12989_2016_149_MOESM2_ESM.pdf]

**Additional file 2: tables**

**Table S1: Oral exposure to Ag NP and mouse final b.w.**

| | | Ag NP concentrations in pellets (ppb) | | | |
|---|---|---|---|---|---|
| experiment | | 0 | 46 | 460 | 4600 |
| | Target intake (µg NP/kg bw/d)[a] | 0 | 11.4 | 114 | 1140 |
| 1 | Pellet consumption (g/d)[b] | 3.1 | 3.9 | 3.3 | 3 |
| | bw (g)[b] | 19.6 ± 0.8 | 19.9 ± 0.26 | 19.9 ± 0.65 | 20.7 ± 0.18 |
| | Calculated intake (µg NP/kg bw/d)[c] | 0 | 8.6 | 71.8 | 679 |
| 2 | Pellet consumption (g/d)[b] | 2.94 | 3.93 | 3.33 | 3.05 |
| | bw (g)[b] | 20.6 ± 0.17 | 21.1 ± 0.35 | 21.6 ± 0.34 | 21.4 ± 0.35 |
| | Calculated intake (µg NP/kg bw/d)[c] | 0 | 8.6 | 71.1 | 654.9 |
| 3 | Pellet consumption (g/d)[b] | 3.64 | 3.74 | 3.55 | 3.83 |
| | bw (g)[b] | 20.7 ± 0.37 | 20.5 ± 0.11 | 20.4 ± 0.35 | 21.2 ± 0.44 |
| | Calculated intake (µg NP/kg bw/d)[c] | 0 | 7.75 | 78.5 | 800.8 |

[a]Based on a mean consumption of 5 g pellets/d for a 20 g-mouse
[b]Averaged over the 28 d exposure period
[c](pellet consumption x Ag NP concentration in pellets)/bw
