# Supplementary material for: Dietary silver nanoparticles can disturb the gut microbiota in mice
Source: Part Fibre Toxicol. 2016 Jul 8;13:38. doi: 10.1186/s12989-016-0149-1 (PMC4939013; doi:10.1186/s12989-016-0149-1)
Supplement: Supplementary file 3 — B.w. and blood C-RP responses in mice orally exposed to Ag NP during 28 d. C57BL/6 female mice were orally exposed to food supplemented with 0, 46, 460 or 4600 ppb nAg during 28 d. (A) B.w. were measured on d 0 prior to first exposure and twice a week during the experimental period. (B) C-RP was quantified in serum by Luminex. Graphs represent means ± SEM (n = 3-5). (PDF 38 kb) [file 12989_2016_149_MOESM3_ESM.pdf]

Additional file 3: figure S1

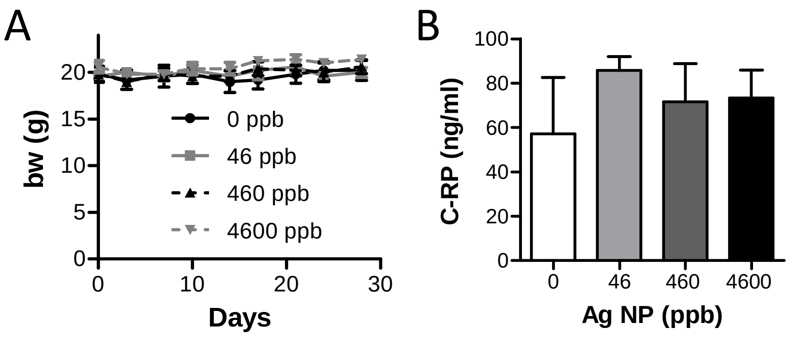

Figure S1: B.w. and blood C-RP responses in mice orally exposed to Ag NP during 28 d. C57BL/6 female mice were orally exposed to food supplemented with 0, 46, 460 or 4600 ppb nAg during 28 d. (A) B.w. were measured on d 0 prior to first exposure and twice a week during the experimental period. (B) C-RP was quantified in serum by Luminex. Graphs represent means ± SEM (n = 3-5).
